# Angebote Früher Hilfen und gesundheitliche Entwicklung von Kindern: Exemplarische Befunde und methodische Herausforderungen bei der Nutzung der Schuleingangsuntersuchung

**DOI:** 10.1007/s00103-024-03955-w

**Published:** 2024-10-06

**Authors:** Simone Weyers, Simon Götz

**Affiliations:** Institut für Medizinische Soziologie, Centre for Health and Society, Medizinische Fakultät und Universitätsklinikum Düsseldorf, Heinrich-Heine-Universität Düsseldorf, Moorenstraße 5, 40225 Düsseldorf, Deutschland

**Keywords:** Gesundheitliche Chancengleichheit, Gesundheitliche Ungleichheit, Prävention, Gesundheitsförderung, Kindergesundheit, Health equity, Health inequality, Prevention, Health promotion, Child health

## Abstract

**Hintergrund:**

Frühe Hilfen sollen Förderangebote systematisch vernetzen und passgenau gestalten, insbesondere für sozioökonomisch benachteiligte Familien. Die Angebote sind universell oder selektiv, die Evidenz zur Wirksamkeit ist jedoch begrenzt.

**Ziele:**

Ziele der Arbeit waren, anhand der Schuleingangsuntersuchungen (SEU) exemplarisch zu untersuchen, ob Teilnehmende an ausgewählten Angeboten der Frühen Hilfen eine bessere Entwicklung haben als Nichtteilnehmende, sowie zu erörtern, inwiefern die SEU genutzt werden kann, um die Wirkung Früher Hilfen zu beurteilen.

**Methode:**

Wir untersuchten 3 typische Angebote der Frühen Hilfen (Familienbildung; Zukunft für Kinder (ZfK); Kita-U) im Zusammenhang mit vollständigem Impfschutz und altersgemäßer Entwicklung bei der U9. Einbezogen wurden die Daten von 4579 Düsseldorfer Schulneulingen. Mittels Propensity-Score-Matching wurden Prozentsatzdifferenzen (Average Treatment Effect on the Treated, ATT) hinsichtlich Impfschutz und Entwicklung zwischen vergleichbaren Interventions- und Kontrollgruppen berechnet.

**Ergebnisse:**

Alle Angebote sind mit einer leicht erhöhten Wahrscheinlichkeit eines vollständigen Impfschutzes verbunden (ATT 2,1 bei Familienbildung; 2,5 bei ZfK; 5,3 bei Kita-U). Familienbildung ist zudem mit einer leicht erhöhten Wahrscheinlichkeit einer altersgemäßen Entwicklung verbunden (ATT 1,6), die Wahrscheinlichkeit einer altersgemäßen Entwicklung bei Teilnehmenden von ZfK (−10,1) und Kita‑U (−4,5) ist geringer.

**Diskussion:**

Die Bewertung Früher Hilfen, insbesondere selektiver Angebote, ist aufgrund von Confounding und geeigneten Vergleichsgruppen eine methodische Herausforderung. Die SEU könnte jedoch unter spezifischen Bedingungen ein Rahmen für Wirkungsanalysen sein.

## Abstract (en)

**Background:**

Early childhood intervention is intended to systematically network and customise support services, particularly for socio-economically disadvantaged families. The programmes are universal or selective, but the evidence on their effectiveness is limited.

**Aims:**

The aims of this study were to exemplary analyse whether participants in early childhood intervention services had better development than non-participants using the school entry examination (SEE) as well as to discuss to what extent the SEE can be used to assess the impact of early childhood intervention services.

**Methods:**

We analysed three typical offers of early childhood intervention services (family education; Zukunft für Kinder (ZfK); Kita-U) in relation to full vaccination coverage and age-appropriate development at U9. Data from 4579 Düsseldorf first graders were included. Propensity score matching was used to calculate percentage differences (average treatment effect on the treated; ATT) in terms of immunisation coverage and development between comparable intervention and control groups.

**Results:**

All programmes are associated with a slightly increased probability of full vaccination protection (ATT 2.1 for family education; 2.5 for ZfK; 5.3 for Kita-U). Family education is also associated with a slightly higher probability of age-appropriate development (ATT 1.6), while the probability of age-appropriate development is lower for participants in ZfK (−10.1) and Kita‑U (−4.5).

**Discussion:**

The evaluation of early childhood intervention, especially selective services, is a methodological challenge due to confounding and suitable comparison groups. However, the SEE could be a framework for impact analyses under specific conditions.

## Einleitung

Sozioökonomisch benachteiligte Kinder haben ein höheres Risiko für Entwicklungsverzögerungen, körperliche und psychische Erkrankungen [1, 2]. Die Entstehung gesundheitlicher Ungleichheiten ist komplex. Daraus ergeben sich vielfältige Ansatzpunkte, um Chancengleichheit in der kindlichen Entwicklung zu fördern [3]. Hierzu existiert eine kommunale Trägerlandschaft mit medizinischen und nichtmedizinischen Angeboten. Allerdings werden diese von sozioökonomisch benachteiligten Kindern und ihren Eltern vergleichsweise selten genutzt, was mit dem Begriff des *Präventionsdilemmas* beschrieben wird [4].

Die Angebote für Zielgruppen systematisch zu verknüpfen, passgenau zu gestalten und dadurch Kinder vor Entwicklungsbeeinträchtigungen zu schützen, ist Aufgabe der Frühen Hilfen. In quartiersbezogenen oder kommunalen Netzwerken der Frühen Hilfen werden Maßnahmen aus unterschiedlichen Sozialsystemen koordiniert und den Familien niedrigschwellig zur Verfügung gestellt [5]. Die Angebote sind universell, d. h. unspezifisch für die Gesamtbevölkerung, oder selektiv, d. h. für Familien mit erhöhtem Beratungs- und Unterstützungsbedarf [6]. Zu den universellen Angeboten zählen z. B. Leistungen der medizinischen Regelversorgung, Willkommensbesuche und Angebote der Familienbildung. Zu den selektiven Angeboten zählen Beratungsstellen und Frühförderung [6] sowie aufsuchende Angebote von Hebammen und Gesundheitsfachkräften.

So auch in Düsseldorf, einer typischen Großstadt, wo folgende Angebote zentrale Bausteine der Frühen Hilfen sind:

(i) *Familienbildung* als universelles Angebot ist ein klassischer Partner von jungen Eltern gemäß Siebtem Buch Sozialgesetzbuch (SGB VIII; [7]). Sie soll Familien bei Erziehungsaufgaben unterstützen und die gesunde Entwicklung der Kinder fördern [8]. Sie vermittelt dazu entsprechendes Wissen und Kompetenzen im Rahmen von Informationsveranstaltungen und Kursen. Durch die Integration der Familienbildung und der Frühen Hilfen ergeben sich neue Zugangswege und Konzepte [7], z. B. in Form bedarfsgerechter oder kostenfreier Angebote im Sozialraum.
(ii) Im selektiven Präventionsprogramm *Zukunft für Kinder (ZfK) *erhalten psychosozial hoch belastete werdende oder junge Eltern interdisziplinäre Beratung und Unterstützung durch Gesundheits- oder sozialpädagogische Fachkräfte. Ziel ist, die Eltern in ihrer Versorgungs- und Erziehungskompetenz zu stärken. Risikolagen sollen früh erkannt werden. Die Beratung erfolgt im häuslichen Umfeld der Familie und bei Bedarf wird sie in weitere Unterstützungsangebote aus Gesundheitshilfe, Jugendhilfe oder dem System sozialer Sicherung begleitet [9].
(iii) Das selektive Angebot *Kita-Eingangsuntersuchung (Kita-U) *richtet sich an Kinder in Kitas in Sozialräumen mit besonderem Handlungsbedarf. Es handelt sich um eine Untersuchung der allgemeinen Entwicklung und des Impf- und Vorsorgestatus – ähnlich der späteren Schuleingangsuntersuchung (SEU). Bei Förderbedarf vermitteln Präventionsmanager:innen an das bestehende Hilfesystem. Das Angebot ist träger- und ämterübergreifend und die Vernetzung von kommunalen Hilfesystemen steht im Vordergrund [10]. Vergleichbare Ansätze gibt es an vielen Standorten in Deutschland [11].

Angebote Früher Hilfen sollen ihre Wirksamkeit unter Beweis stellen. Dies gilt sowohl fachlich im Sinne positiver Effekte auf die Klient:innen als auch verwaltungstechnisch im Zuge der Steuerung öffentlicher Leistungen [12]. Zu den Auswirkungen oben genannter oder vergleichbarer Angebote auf die kindliche Entwicklung gibt es in der Literatur eine überschaubare Anzahl von Arbeiten.

### Zu Punkt (i):

Bei der Bewertung der universellen *Familienbildung* steht die sozioemotionale Entwicklung im Fokus. So belegen beispielsweise Li et al. kleinere Effekte des Programmes *Tripple P *[13]. Weiss et al. zeigen, dass das sozioemotionale Verhalten der Kinder durch Familienbildungsprogramme in kleinem Umfang positiv beeinflusst werden kann [14]. Arbeiten zu den Auswirkungen auf die körperliche Entwicklung gibt es unseres Wissens nicht. Angesichts des breiten Angebots an Familienbildung in der Bundesrepublik ist die Anzahl der Evaluationsstudien gering [14].

### Zu Punkt (ii):

Das selektive Angebot ZfK wurde anhand der Angaben von Fachkräften evaluiert. Verglichen wurden dabei die Angaben zur Entwicklung der Kinder infolge des Angebotes mit Angaben zur Entwicklung von Kindern, die durch die Jugendhilfe betreut wurden. Die Fachkräfte von *ZfK* berichteten positive Veränderungen in der sozioemotionalen und körperlichen Entwicklung der Kinder, nicht jedoch die Fachkräfte in der Vergleichsgruppe [15]. Die Autor:innen schlussfolgern, dass belastete Familien durch das Präventionsprogramm früher erreicht und begleitet werden. Objektive Daten wurden in diese Evaluation jedoch nicht einbezogen. Eine ältere Metaanalyse [16] identifizierte 8 vergleichbare Angebote, die standardisierte Tests heranzogen, bei denen sich insgesamt ein Programmeffekt nahe null auf die psychische Entwicklung und ein Nulleffekt auf die körperliche Entwicklung zeigten. Vereinzelt weisen neuere Auswertungen auf einen moderaten Effekt von Gesundheitsfachkräften auf die sozioemotionale Entwicklung, nicht jedoch auf die körperliche Entwicklung der Kinder hin (z. B. [17]).

### Zu Punkt (iii):

Zur Bewertung von Angeboten, die der *Kita‑U* vergleichbar sind, wurden Daten der SEU genutzt. In Sachsen [18] und Hildesheim [19] wurde gezeigt, dass Kinder mit zuvor erfolgter Untersuchung in der Kita bei der SEU besser entwickelt waren als Kinder ohne Untersuchung in der Kita. Mit der SEU stehen ärztliche Routinedaten zur Entwicklung und Gesundheit der Kinder aller Bevölkerungsgruppen zur Verfügung, die zur Bewertung von Angeboten der Frühen Hilfen genutzt werden könnten. Bisher gibt es allerdings nur wenige Beispiele.

Angesichts der begrenzten Datenlage ist das Ziel der vorliegenden Arbeit, erstens im Rahmen der SEU exemplarisch zu prüfen, ob Kinder, die selbst oder über ihre Eltern an den Angeboten teilgenommen haben, eine bessere Entwicklung bei Schuleingang haben als vergleichbare Nichtteilnehmende. Auf Basis dieser Zusammenhangsanalyse möchten wir zweitens erörtern, inwiefern die SEU grundsätzlich genutzt werden könnte, um die Wirkung von Angeboten der Frühen Hilfen zu beurteilen.

## Methoden

Die Arbeit basiert auf den Daten der retrospektiven Kohortenstudie *Gesundheit bei Schuleingang* [20]. Hauptziel der Studie war zu prüfen, ob Präventionsnutzung mit einer besseren Entwicklung bei Schuleingang verbunden ist. Hierzu wurden Untersuchungsdaten von Düsseldorfer Schulneulingen mit Elternangaben zur Nutzung von Präventionsangeboten kombiniert. In Düsseldorf werden jährlich ca. 5000 Schulneulinge vom Kinderärztlichen Jugend- und Gesundheitsdienst untersucht, um schulrelevante Entwicklungsstörungen zu erkennen und die Eltern zu Fördermaßnahmen zu beraten. Die Elternbefragung fand während der SEU im Gesundheitsamt statt. Dort wurden die Eltern aller Schulneulinge 2017 und 2018 zu einer standardisierten schriftlichen Befragung bzgl. ihrer Teilnahme an Präventionsangeboten bis Schuleingang eingeladen. Es bestanden keine Zugangsvoraussetzungen. Fragebögen wurden in 6 Sprachen vorgehalten. Für die Teilnahme wurde keine Gratifikation gewährt. Insgesamt wurden 6480 Vorschulkinder in die Studie eingeschlossen, deren Befragungsdaten durch ein Pseudonym mit den amtsärztlichen Untersuchungsdaten verbunden wurden.

Die für die vorliegende Fragestellung relevanten Variablen wurden folgendermaßen erhoben und operationalisiert. Exposition: Die Teilnahme an den Angeboten der Frühen Hilfen (*Familienbildung, ZfK, Kita‑U*) wurde mit dem standardisierten Elternfragebogen erhoben. Für die Analysen wurden Angebotsteilnehmende den Nichtteilnehmenden gegenübergestellt. Elternangaben wurden nötigenfalls mit den Daten des Gesundheitsamtes abgeglichen.

Outcome: Wir wählten exemplarisch 2 Variablen, die in der SEU routinemäßig erfasst werden und bedeutsam für die kindliche Entwicklung sind: Der vollständige *Impfschutz* schützt vor infektionsbedingten Wachstumsverzögerungen [21] und wurde in der SEU anhand des Impfpasses erhoben. Die *altersgemäße* (motorische, sprachliche und sozioemotionale) *Entwicklung* mit 5 Jahren wurde in der Früherkennungsuntersuchung U9 dokumentiert und anhand des gelben Untersuchungshefts erhoben. Vergleichsgruppe waren jeweils Kinder ohne vollständigen Impfschutz bzw. mit nicht altersgemäßer Entwicklung bei der U9.

Confounder: Ein Confounder ist ein Risikofaktor für die interessierende Zielgröße, der mit der interessierenden Exposition assoziiert ist und nicht in der Kausalkette zwischen Exposition und Zielgröße steht. Wird diese Assoziation in der Auswertung nicht berücksichtigt, führt dies zu einer verzerrten Schätzung des Effekts der untersuchten Exposition [22]. Wichtige Confounder bei der hier vorliegenden Fragestellung sind Merkmale der sozialen Lage, da diese sowohl mit der Nutzung von Angeboten der Frühen Hilfen [23] als auch mit der kindlichen Entwicklung [24] assoziiert sind. Wir schlossen folgende Confounder ein: Die *elterliche Bildung* wurde in der Elternbefragung erhoben und nach CASMIN(Comparative Analysis of Social Mobility in Industrial Nations)-Klassifikation [25] in niedrig (Familien mit max. Hauptschulabschluss), mittel (max. Abitur mit Lehrausbildung) und hoch (Hochschulabschluss) unterschieden. Folgende Merkmale wurden in der SEU erhoben: Der *sozialräumliche Belastungsgrad* im Quartier hat 5 Kategorien (sehr hoher, hoher, mittlerer, geringer und sehr geringer Belastungsgrad). Beim *Familienstatus* wurden Einelternfamilien mit Zweielternfamilien verglichen. *Migrationshintergrund* lag vor, wenn mindestens ein Elternteil nicht in Deutschland geboren ist, Vergleichsgruppe waren Kinder, deren Eltern beide in Deutschland geboren sind. Beim *Geschlecht* unterschieden wir Jungen und Mädchen. Bei den selektiven Angeboten für besonders belastete Familien wurden zusätzlich *psychosoziale Belastungen bei Geburt* (alleinige Verantwortung für Kinder, berufliche Situation oder Arbeitslosigkeit, finanzielle Sorgen, Konflikte mit dem (Ex‑)Partner) berücksichtigt und in (sehr) stark belastet vs. mittel oder (sehr) wenig belastet unterschieden. Dieses Merkmal wurde ebenfalls in der Elternbefragung erfasst.

Analyse: Um hinsichtlich der beobachteten Confounder möglichst vergleichbare Gruppen zu bekommen, führten wir ein *Propensity-Score-Matching* [26, 27] durch. Diese Methode ist besonders geeignet Behandlungseffekte zu schätzen, wenn sich Behandelte und Nichtbehandelte in ihren Eigenschaften extrem unterschieden. Dies trifft in der vorliegenden Analyse insbesondere auf die selektiven Angebote zu [27]. In einem ersten Schritt wurde mithilfe eines logistischen Regressionsmodells die Wahrscheinlichkeit (Propensity-Score) für jede Person geschätzt, zur Interventionsgruppe zu gehören. Dazu sollten möglichst alle Variablen genutzt werden, die sowohl für die Teilnahme als auch für das Outcome relevant sind [28]. Für die vorliegende Fragestellung wurden alle 6 oben genannten Confounder berücksichtigt, mit Ausnahme der psychosozialen Belastungen bei Geburt für *Familienbildung*, da sich diese an alle Eltern richtet. Anschließend wurde mithilfe eines Matching-Algorithmus der Interventionsgruppe eine Kontrollgruppe zugewiesen, die in ihrem Propensity-Score und damit in ihrer Zusammensetzung bezüglich möglicher Confounder ähnlich ist, die Intervention aber nicht bekommen hat. In der vorliegenden Analyse kam ein Kernel-Matching („Epanechnikov’s kernel function“) zum Einsatz, da diese Technik im Gegensatz zu anderen Matching-Methoden mehr verfügbare Informationen für die Analyse nutzbar macht und sich dadurch als effizient im Umgang mit den verfügbaren Daten erwiesen hat. Bei anderen Matching-Methoden, wie beispielsweise dem 1:1-Matching, würden viele mögliche Kontrollen nicht berücksichtigt werden, da jeder Beobachtung in der Treatment-Gruppe nur eine Kontrolle zugeordnet wird. [29]. Bei *Kita‑U* wurde bzgl. des sozialräumlichen Belastungsgrades statt des Matchings über den Propensity-Score ein direktes Matching [30] genutzt, um nur Kinder innerhalb des Belastungsgrades zu vergleichen, da die *Kita‑U* in sozialräumlich belasteten Stadtteilen durchgeführt wird. Die Analysen wurden mit dem Modul KMATCH in Stata18 durchgeführt [31].

Dann wurde die Häufigkeit der Merkmalsausprägungen in Interventions- und Kontrollgruppe vor und nach dem Matching verglichen. Damit lässt sich prüfen, ob durch das Matching eine ähnliche Verteilung der Confounder zwischen der Interventions- und Kontrollgruppe hergestellt werden konnte.

Schließlich wurden die Wahrscheinlichkeiten der Outcomes zwischen beiden Gruppen verglichen. Die Prozentsatzdifferenz (Average Treatment Effect on the Treated, ATT) beschreibt bei Berücksichtigung aller relevanten Variablen den durchschnittlichen Effekt einer Intervention auf diejenigen, die an der Intervention teilgenommen haben [28]. Konfidenzintervalle wurden mittels Bootstrapping ermittelt, um die Effekte auf statistische Signifikanz zu testen [30].

## Ergebnisse

Von den insgesamt 9894 untersuchten Kindern lagen von 6480 Eltern Fragebögen vor, die mit den Untersuchungsdaten verbunden werden konnten (Rücklauf 65,5 %). In die vorliegende Analyse wurden 4579 Kinder mit vollständigen Daten einbezogen. Sozial belastete Personen sind im Analysesample unterrepräsentiert: Je niedriger die Bildung oder der sozialräumliche Belastungsgrad, desto häufiger fehlen Werte. Wir gehen davon aus, dass dies unsere Ergebnisse nicht wesentlich beeinflusst, da die Verteilung der fehlenden Werte nach Teilnahme und Nichtteilnahme nicht systematisch variieren. Tab. 1 ist zu entnehmen, dass 50,6 % der Eltern an *Familienbildung* teilnahmen, 1,5 % an *ZfK* und 0,9 % an *Kita‑U*. Bei 82,0 % der Kinder bestand ein vollständiger Impfschutz und 80,4 % waren altersgemäß entwickelt.

**Tab. 1:** Stichprobenbeschreibung

Variablen (fehlende Werte)	*n*	%
*Teilnahme Familienbildung (208)*		
Nein	2262	49,4
Ja	2317	50,6
*Teilnahme Zukunft für Kinder (244)*		
Nein	4512	98,5
Ja	67	1,5
*Teilnahme Kita‑U (0)*		
Nein	4539	99,1
Ja	40	0,9
*Geschlecht des Kindes (3)*		
Weiblich	2217	48,4
Männlich	2362	51,6
*Elterliche Bildung (386)*		
Hoch	2693	58,8
Mittel	1524	33,3
Niedrig	362	7,9
*Sozialräumlicher Belastungsgrad (112)*		
Sehr gering	865	18,9
Gering	1392	30,4
Mittel	1287	28,1
Hoch	820	17,9
Sehr hoch	215	4,7
*Familienstatus (0)*		
Zweielternfamilien	4013	87,6
Einelternfamilien	566	12,4
*Migrationshintergrund (416)*		
Nein	2386	52,1
Ja	2193	47,9
*Belastungen Geburt: alleinige Verantwortung für Kinder (747)*		
Nein/gering	4396	96,0
(Sehr) stark	183	4,0
*Belastungen Geburt: berufliche Situation, Arbeitslosigkeit (768)*		
Nein/gering	4319	94,3
(Sehr) stark	260	5,7
*Belastungen Geburt: finanzielle Sorgen (712)*		
Nein/gering	4330	94,6
(Sehr) stark	249	5,4
*Belastungen Geburt: Konflikte mit dem (Ex‑)Partner (756)*		
Nein/gering	4363	95,3
(Sehr) stark	216	4,7
*Vollständiger Impfschutz (565)*		
Nein	822	18,0
Ja	3757	82,0
*Altersgemäße Entwicklung (0)*		
Nein	899	19,6
Ja	3680	80,4
Total	4579	100,0

Einen ersten Hinweis zu unserer Forschungsfrage gibt Tab. 2. Sie zeigt die unadjustierten absoluten und relativen Häufigkeiten der beiden Outcomes und möglichen Confounder bei den jeweiligen Angebotsteilnehmenden und Nichtteilnehmenden. Bei Angebotsteilnehmenden zeigt sich eine erhöhte Wahrscheinlichkeit eines vollständigen Impfschutzes gegenüber Nichtteilnehmenden (*Familienbildung* 83,8 % vs. 80,3 %; *ZfK* 85,1 % vs. 82,0 %; *Kita‑U* 87,5 % vs. 82,0 %). Anders sieht es bei der altersgemäßen Entwicklung aus: Hier haben Teilnehmende der universellen Elternbildung eine höhere Wahrscheinlichkeit einer altersgemäßen Entwicklung (84,2 % vs. 76,5 %), Teilnehmende der selektiven Angebote jedoch eine geringere Wahrscheinlichkeit einer altersgemäßen Entwicklung (*ZfK* 67,2 % vs. 80,6 %; *Kita‑U* 70,0 % vs. 80,5 %).

**Tab. 2:** Bivariate Analysen: absolute (*n*) und relative (%) Häufigkeiten der Ergebnisse und der potenziellen Confounder nach Teilnahme an den Angeboten der Frühen Hilfen

	Familienbildung				Zukunft für Kinder				Kita‑U			
	Ja		Nein		Ja		Nein		Ja		Nein	
	*n*	%	*n*	%	*n*	%	*n*	%	*n*	%	*n*	%
*Vollständiger Impfschutz*												
Nein	376	16,2	446	19,7	10	14,9	812	18,0	5	12,5	817	18,0
Ja	1941	83,8	1816	80,3	57	85,1	3700	82,0	35	87,5	3722	82,0
*Altersgemäße Entwicklung*												
Nein	367	15,8	532	23,5	22	32,8	877	19,4	12	30,0	887	19,5
Ja	1950	84,2	1730	76,5	45	67,2	3635	80,6	28	70,0	3652	80,5
*Geschlecht des Kindes*												
Weiblich	1107	47,8	1110	49,1	30	44,8	2187	48,5	18	45,0	2199	48,4
Männlich	1210	52,2	1152	50,9	37	55,2	2325	51,5	22	55,0	2340	51,6
*Elterliche Bildung*												
Hoch	1663	71,8	1030	45,5	23	34,3	2670	59,2	7	17,5	2686	59,2
Mittel	596	25,7	928	41,0	35	52,2	1489	33,0	21	52,5	1503	33,1
Niedrig	58	2,5	304	13,4	9	13,4	353	7,8	12	30,0	350	7,7
*Sozialräumlicher Belastungsgrad*												
Sehr gering	512	22,1	353	15,6	9	13,4	856	19,0	0	0,0	865	19,1
Gering	831	35,9	561	24,8	12	17,9	1380	30,6	5	12,5	1387	30,6
Mittel	620	26,8	667	29,5	15	22,4	1272	28,2	6	15,0	1281	28,2
Hoch	302	13,0	518	22,9	22	32,8	798	17,7	16	40,0	804	17,7
Sehr hoch	52	2,2	163	7,2	9	13,4	206	4,6	13	32,5	202	4,5
*Familienstatus*												
Zweielternfamilien	2110	91,1	1903	84,1	47	70,1	3966	87,9	33	82,5	3980	87,7
Einelternfamilien	207	8,9	359	15,9	20	29,9	546	12,1	7	17,5	559	12,3
*Migrationshintergrund*												
Nein	1525	65,8	861	38,1	31	46,3	2355	52,2	10	25,0	2376	52,3
Ja	792	34,2	1401	61,9	36	53,7	2157	47,8	30	75,0	2163	47,7
*BG: alleinige Verantwortung Kinder*												
Nein/gering	2247	97,0	2149	95,0	60	89,6	4336	96,1	38	95,0	4358	96,0
(Sehr) stark	70	3,0	113	5,0	7	10,4	176	3,9	2	5,0	181	4,0
*BG: berufliche Situation, Arbeitslosigkeit*												
Nein/gering	2212	95,5	2107	93,1	62	92,5	4257	94,3	36	90,0	4283	94,4
(Sehr) stark	105	4,5	155	6,9	5	7,5	255	5,7	4	10,0	256	5,6
*BG: finanzielle Sorgen*												
Nein/gering	2227	96,1	2103	93,0	60	89,6	4270	94,6	37	92,5	4293	94,6
(Sehr) stark	90	3,9	159	7,0	7	10,4	242	5,4	3	7,5	246	5,4
*BG: Konflikte mit dem (Ex‑)Partner*												
Nein/gering	2236	96,5	2127	94	61	91,0	4302	95,3	39	97,5	4324	95,3
(Sehr) stark	81	3,5	135	6	6	9,0	210	4,7	1	2,5	215	4,7
Total	2317	100,0	2262	100,0	67	100,0	4512	100,0	40	100,0	4539	100

*BG* Belastungen bei Geburt

Allerdings unterscheiden sich die Angebotsteilnehmenden der Frühen Hilfen auch bezüglich der Confounder. Erwartungsgemäß sind in den selektiven Angeboten insbesondere vulnerable Personen zu finden, und zwar Kinder mit niedriger Elternbildung und Kinder von Einelternfamilien. Teilnehmende von *ZfK* hatten häufiger Belastungen bei Geburt, Teilnehmende der *Kita‑U* sind insbesondere aus belasteten Sozialräumen. Bei den Teilnehmenden der universellen *Familienbildung* zeigt sich ein Bild, wie es oft bei der Nutzung von Präventionsangeboten zu beobachten ist (Präventionsdilemma). Die Angebote werden überdurchschnittlich häufig von Familien mit hoher elterlicher Bildung, Familien ohne Migrationshintergrund, Zweielternfamilien und Familien mit wenigen psychosozialen Belastungen zur Zeit der Geburt genutzt. Dies macht die besondere Herausforderung in der Wirkungsanalyse von Angeboten der Frühen Hilfen deutlich. Die Gruppen unterscheiden sich also sowohl in ihrer Teilnahme als auch in den Gesundheitsdeterminanten, hier: Confoundern.

Tab. 3 zeigt den Vergleich der Teilnehmenden („treated“) und Nichtteilnehmenden vor („raw untreated“) und nach dem Propensity-Score-Matching („matched untreated“). Beispielsweise kamen bei der *Familienbildung* vor dem Matching 2,5 % der Teilnehmenden, jedoch 13,4 % der Nichtteilnehmenden aus einem Haushalt mit niedriger Bildung, nach dem Matching beträgt der Anteil unter den Nichtteilnehmenden nur noch 3,0 %. Das Potenzial zur Verzerrung durch Unterschiede im Bildungsniveau ist also reduziert. Insgesamt zeigt Tab. 3, dass die Gruppen der Teilnehmenden und der Nichtteilnehmenden bezüglich der Verteilung der Confounder deutlich ähnlicher geworden sind. Im Falle der *Kita‑U* sind die Anteilswerte bezüglich des Sozialraumes gleich, da hier aufgrund der Bedeutung des Sozialraumes für die Teilnahme ein direktes Matching durchgeführt wurde.

**Tab. 3:** Relative Häufigkeiten der Merkmalsausprägungen (%) der Confounder der Interventionsgruppe („treated“) und der Kontrollgruppe vor („raw untreated“) und nach dem Matching („matched untreated“)

	Familienbildung			Zukunft für Kinder			Kita‑U		
	Treated	Raw	Matched	Treated	Raw	Matched	Treated	Raw	Matched
		Untreated	Untreated		Untreated	Untreated		Untreated	Untreated
Weiblich	47,8	49,1	48,0	44,8	48,5	44,3	45,0	48,4	47,5
Männlich	52,2	50,9	52,0	55,2	51,5	55,7	55,0	51,6	52,5
Hohe elterliche Bildung	71,8	45,6	72,0	34,4	59,2	36,6	17,5	59,2	28,1
Mittlere elterliche Bildung	25,7	41,0	25,0	52,2	33,0	50,3	52,5	33,1	47,3
Niedrige elterliche Bildung	2,5	13,4	3,0	13,4	7,8	13,1	30,0	7,7	24,6
Sehr geringer sozialräumlicher Belastungsgrad	22,1	15,6	22,2	13,5	18,9	13,7	0	19,0	0
Geringer sozialräumlicher Belastungsgrad	35,9	24,8	36,8	17,9	30,6	19,4	12,5	30,6	12,5
Mittlerer sozialräumlicher Belastungsgrad	26,8	29,5	26,2	22,4	28,2	22,7	15,0	28,2	15,0
Hoher sozialräumlicher Belastungsgrad	13,0	22,9	12,3	32,8	17,7	31,5	40,0	17,7	40,0
Sehr hoher sozialräumlicher Belastungsgrad	2,2	7,2	2,5	13,4	4,6	12,7	32,5	4,5	32,5
Mehrelternfamilien	91,1	84,1	90,4	70,1	87,9	72,6	82,5	87,7	81,3
Einelternfamilien	8,9	15,9	9,6	29,9	12,1	27,4	17,5	12,3	18,7
Kein Migrationshintergrund	65,8	38,1	66,2	46,3	52,2	46,8	25,0	52,3	31,8
Migrationshintergrund	34,2	61,9	33,8	53,7	47,8	53,2	75,0	47,7	68,2
BG: alleinige Verantwortung für Kinder	–	–	–	10,4	3,9	10,0	5,0	4,0	4,8
BG: berufliche Situation, Arbeitslosigkeit	–	–	–	7,5	5,7	6,5	10,0	5,6	7,4
BG: finanzielle Sorgen	–	–	–	10,4	5,4	9,6	7,5	5,4	7,3
BG: Konflikte mit dem (Ex‑)Partner	–	–	–	9,0	4,7	9,0	2,5	4,7	5,0

*BG* Belastungen bei Geburt

Im letzten Schritt wird die Differenz zwischen den Wahrscheinlichkeiten eines vollständigen Impfschutzes bzw. einer altersgemäßen Entwicklung zwischen den Teilnehmenden und gematchten Nichtteilnehmenden berechnet (Tab. 4). Diese Differenz steht für den durchschnittlichen Effekt einer Intervention auf diejenigen, die an der Intervention teilgenommen haben (ATT). Für alle Angebote zeigt sich eine höhere Wahrscheinlichkeit eines vollständigen Impfschutzes (2,1 Prozentpunkte bei *Familienbildung*; 2,5 bei *ZfK*; 5,3 bei *Kita‑U*). Bei *Familienbildung* zeigt sich eine leicht erhöhte Wahrscheinlichkeit (1,6 Prozentpunkte) einer altersgemäßen Entwicklung, bei den selektiven Angeboten zeigt sich eine geringere Wahrscheinlichkeit (−10,1 Prozentpunkte bei *ZfK*; −4,5 bei *Kita‑U*). Die Ergebnisse sind statistisch nicht signifikant. Bei den selektiven Angeboten sind die Konfidenzintervalle aufgrund der geringen Fallzahlen (*ZfK: n* = 67; *Kita-U: n* = 40) erwartungsgemäß breit.

**Tab. 4:** Treatment-Effekte

	Familienbildung			Zukunft für Kinder			Kita‑U		
	ATT	95 % KI		ATT	95 % KI		ATT	95 % KI	
Vollständiger Impfschutz	2,1	−0,4	4,6	2,5	−6,3	11,3	5,3	−5,7	16,4
Altersgemäße Entwicklung	1,6	−0,7	3,8	−10,1	−22,4	2,2	−4,5	−20,4	11,5

*ATT* Average Treatment Effect on the Treated; *KI* Konfidenzintervall

## Diskussion

Ziel der vorliegenden Arbeit war erstens, im Rahmen der SEU exemplarisch zu prüfen, ob Kinder, die selbst oder über ihre Eltern an Angeboten der Frühen Hilfen teilgenommen haben, eine bessere Entwicklung bei Schuleingang haben als vergleichbare Nichtteilnehmende. Zusammengefasst zeigt sich bei allen 3 Angeboten, dass Teilnehmende jeweils eine leicht erhöhte Wahrscheinlichkeit eines vollständigen Impfschutzes aufweisen. Bei Teilnehmenden der *Familienbildung* zeigt sich zudem eine leicht erhöhte Wahrscheinlichkeit einer altersgemäßen Entwicklung, während die Wahrscheinlichkeit einer altersgemäßen Entwicklung bei Teilnehmenden von *ZfK* und *Kita‑U* geringer ist.

Der positive Zusammenhang der universellen *Familienbildung* mit der altersgemäßen Entwicklung entspricht bisherigen Studien zur sozioemotionalen Entwicklung [13, 14], er ist jedoch geringer. Evidenz zum Impfschutz gibt es in diesem Bereich unseres Ermessens bisher nicht.

Der negative Zusammenhang von *Kita‑U* und altersgemäßer Entwicklung entspricht nicht der Evidenz zu den sächsischen *Kita‑U*-Kindern. Diese erhielten in der SEU seltener Arztüberweisungen in den Entwicklungsbereichen Sprache, Motorik, Sehen und Hören als Kinder ohne *Kita‑U* [18]. Allerdings ist unklar, inwiefern Interventions- und Kontrollgruppe vergleichbar waren. Der positive Zusammenhang von *Kita‑U* und vollständigem Impfschutz hingegen entspricht der Studie zu den Hildesheimer Kindern. Dort wurden explizit Kinder aus sozialräumlich belasteten Quartieren mit und ohne *PIAF* (*Prävention in aller Frühe, *ähnlich *Kita‑U*) verglichen. Dabei zeigte sich, dass die *PIAF*-Kinder häufiger vollständig geimpft waren als die Kontrollgruppe [19].

Der negative Zusammenhang von *ZfK* und altersgemäßer Entwicklung steht nicht im Widerspruch zum Nulleffekt, den Taubner et al. [16] in ihrer Metaanalyse finden. Die Autor:innen führen den Effekt auf eine verzerrte Stichprobe zurück. Selektive Programme stünden vor der Herausforderung, Veränderungen für eine stark belastete Klientel während einer begrenzten Programmlaufzeit zu realisieren. Deshalb sind bessere Ergebnisse nicht zu erwarten. Dies trifft auf *ZfK* in besonderer Weise zu, da sich das Angebot an Eltern in einer sehr stark belasteten Situation richtet, die geprägt ist durch Armut, Sucht, chronische Erkrankungen oder Gewalt. Da dieser stark selektiven Zielgruppe aufgrund fehlender Informationen keine adäquate Kontrollgruppe gegenübergestellt werden kann, ist bei *Zfk* ein negativer Zusammenhang mit der kindlichen Entwicklung auch mit einer positiven Auswirkung des Programms vereinbar.

Das Problem der selektiven Zielgruppe führt zur methodischen Problematik der fehlenden Vergleichsgruppe – und damit zum zweiten Ziel der vorliegenden Arbeit. Zu erörtern war, inwiefern die SEU grundsätzlich genutzt werden könnte, um die Wirkung universeller und selektiver Angebote der Frühen Hilfen zu beurteilen. In unserer Analyse wurde eine entlang der vorhandenen Confounder weitgehend vergleichbare Kontrollgruppe durch Matching gebildet. Confounder bei der vorliegenden Fragestellung sind Merkmale der sozialen Lage, da diese sowohl mit der Angebotsnutzung als auch mit der kindlichen Entwicklung assoziiert sind. Einige dieser Merkmale konnten wir einschließen, weil sie in der Elternbefragung erhoben wurden. Regulär fehlen sie jedoch oft in der SEU, denn sie werden von vielen Kommunen nicht automatisch erhoben [32]. Da es sich bei der Genese von Entwicklungsproblemen im Kindesalter um ein multifaktorielles Geschehen handelt, kommen weitere Confounder infrage, wie z. B. die Nutzung sonstiger Förderangebote, das Gesundheitsverhalten, die Wohnverhältnisse oder die Infrastruktur im Quartier. Für unsere Analyse lagen solche Merkmale nicht vor.

Die allgemeine Herausforderung einer Wirkungsanalyse von selektiven Angeboten geht jedoch über das hinaus, was mit den Variablen, die standardmäßig als Confounder erhoben werden, kontrolliert werden kann. Bei der Zielgruppe von *ZfK* handelt es sich um Familien in hoch problematischen Lebenssituationen. Die uns vorliegenden Informationen reichen nicht aus, um solche Probleme vollständig abzubilden und im statistischen Matching vergleichbare Gruppen zu bilden.

Die Problematik der fehlenden Vergleichsgruppe wurde bereits in der Rehabilitationsforschung diskutiert [33]. Eine für die Wirkungsanalyse Früher Hilfen interessante Option scheinen uns historische Kontrollen (d. h. Gruppen vor Einführung einer neuen Maßnahme) zu sein, sofern für diese ausreichende Informationen zu Intervention, Behandlungsergebnis und potenziellen Confoundern vorliegen. Die SEU mit ihren jährlichen Kohorten sind eine gute Datengrundlage. So konnte beispielsweise in einer Trendstudie gezeigt werden, dass die Teilnahme an der U9 nach Einführung eines Kontroll- und Erinnerungssystems angestiegen ist, insbesondere bei sozioökonomisch benachteiligten Kindern [34]. In ähnlicher Weise könnten in der SEU gesundheitsbezogene Merkmale von Kindern vor Einführung der *Kita‑U* mit den Merkmalen von Kindern nach der Einführung verglichen werden.

Ebenfalls aus der Rehabilitationsforschung kommt ein Vorschlag, wie sich in Abwesenheit einer Vergleichsgruppe eine Veränderung bei Zielgrößen abbilden lässt: Hier ist zum einen der klassische Prä-Post-Ansatz zu nennen, bei dem das Veränderungsmaß in der Interventionsgruppe durch die Differenz zwischen 2 Messungen gewonnen wird. Alternativ ermöglichen Quasivarianten, nur die Postmessung vorzunehmen und die Prämessung retrospektiv aus dem Gedächtnis zu erheben oder Probanden nur die erlebte Veränderung postinterventionell beurteilen zu lassen [35]. Mit diesen deutlich ökonomischeren Verfahren könnten auch Veränderungen (wohlgemerkt: nicht Wirkungen) nach der Nutzung von Angeboten der Frühen Hilfen aufgezeigt werden, z. B. indem die Untersuchungsdaten der SEU als Postmessung genutzt werden und die Eltern retrospektiv den Zustand vor dem Angebot berichten. Alternativ könnten Eltern in der SEU subjektiv die Veränderungen nach der Nutzung von Angeboten berichten, z. B. mit standardisierter Befragung.

### Limitationen

Eine zu den genannten methodischen Problemen hinzukommende Limitation unserer Analyse ist, dass Informationen zur Teilnahme an Frühen Hilfen auf retrospektiven Elternangaben basieren. Hier können Erinnerungsfehler nicht ausgeschlossen werden. Zudem ist die Auswahl von 2 Indikatoren der kindlichen Entwicklung aufgrund des exemplarischen Charakters unserer Studie begrenzt. Eine Stärke ist allerdings, dass sie auf einer ärztlichen Messung beruhen. Eine weitere Limitation ist die geringe Fallzahl selektiver Angebote, weswegen keine robuste statistische Analyse möglich ist. Dies wird durch die fehlende statistische Signifikanz der Effekte deutlich. Dadurch können wir nicht ausschließen, dass unsere Ergebnisse auf Zufallsfehler zurückzuführen sind.

Unter Berücksichtigung der vorangehenden Punkte möchten wir hinsichtlich des zweiten Studienziels festhalten, dass die SEU u. E. unter bestimmten Bedingungen dazu genutzt werden kann, Hinweise auf die Wirkung Früher Hilfen zu erhalten. Hier sind verschiedene Aspekte zu überlegen:

#### Exposition:

Je nach Fragestellung muss die vorherige Teilnahme am Angebot der Frühen Hilfen erhoben werden, durch Elternbefragung oder – um Erinnerungsfehler zu vermeiden – durch Dokumentation der Angebotsträger. Bei der Verknüpfung von Daten aus verschiedenen Abteilungen oder Ämtern (Daten-Linkage) müssen Datenschutzvorgaben berücksichtigt werden.

#### Outcome:

Die SEU produziert eine Reihe objektiver Daten zur Kindesentwicklung. Hier müssen je nach Zielkriterien der untersuchten Angebote geeignete Outcomes ausgewählt werden. Standardmäßig werden Sprache, Motorik, Body-Mass-Index etc. erhoben. Die sozioemotionale Entwicklung des Kindes wird jedoch nicht in allen Kommunen systematisch gemessen. Bei Bedarf stehen validierte Instrumente zur Verfügung, wie z. B. der Strengths and Difficulties Questionniare [36]. Zudem ist die Qualität der Routinedaten trotz Standardverfahren (z. B. SOPESS [37]) nicht unumstritten [38, 39].

#### Confounding:

Die SEU schließt Kinder aus allen Teilen der Bevölkerung ein und erlaubt im Prinzip, vergleichbare Gruppen zu bilden. Hierzu müssen allerdings die nötigen Daten erhoben werden. Angaben zur sozialen Lage werden nicht in allen Kommunen systematisch gemessen. Auch hier stehen standardisierte Instrumente zur Verfügung, wie z. B. der Brandenburger Sozialindex [40]. Bei dieser Gelegenheit können auch weitere für die jeweilige Fragestellung relevante Confounder erhoben werden, wie z. B. psychosoziale Probleme im Falle selektiver Angebote (zum Überblick entsprechender Erhebungsinstrumente [41]).

#### Analyse:

Das Propensity-Score-Matching bietet die Möglichkeit, dem Design einer randomisierten Studie mit Beobachtungsdaten nahe zu kommen. Dies trifft jedoch nur auf bekannte und tatsächlich gemessene Merkmale zu. Damit bei geringen Fallzahlen statistisch robuste Ergebnisse produziert werden können, könnten Daten mehrerer Jahrgänge oder auch Kommunen verbunden (gepoolt) werden. Vielversprechend in diesem Zusammenhang sind aktuelle Entwicklungen hin zu einem überregionalen *Netzwerk Öffentliches Gesundheitswesen*, wie es derzeit in Nordrhein-Westfalen der Fall ist. Für all dies braucht es freilich Ressourcen für den Kinder- und Jugendgesundheitsdienst.

Zum Schluss möchten wir 2 Dinge thematisieren: Auch bei Berücksichtigung der oben genannten Aspekte zur Nutzung der SEU kann erstens Confounding nicht vollständig vermieden werden. Der Ausschluss aller Störvariablen gelingt nur durch randomisierte kontrollierte Studien (RCT), die als der Goldstandard der Wirkungsforschung gelten [42]. Abgesehen davon, dass bei natürlich verlaufenden Expositionen wie der Angebotsteilnahme eine RCT wenig praktikabel ist, gibt es Zweifel, inwiefern die hohe interne Validität solcher Studienergebnisse der individuellen Situation von Zielpersonen gerecht wird [43]. Es wird kritisiert, dass eine Kausalbeschreibung von RCTs eine Standardisierung der Kinder- und Jugendhilfepraxis impliziert, die nicht standardisierbar ist [12]. Ein Teil der Wirkungsvarianz der Jugendhilfe wird durch Partizipation und Arbeitsbeziehung erklärt (siehe auch [44]). Es wird daher auch für die Erforschung von Bedingungen plädiert, unter denen Ursache-Wirkungs-Zusammenhänge entstehen, und von Evidenz, welches Vorgehen mit Blick auf die je fallspezifische Konstellation angemessen ist [12].

Zweitens lohnt bei der Frage nach Wirkung ein Blick auf die *Kausalitätskriterien* von Bradford Hill [45]. Dieser hat eine Reihe von Punkten formuliert, wann auch ohne Experiment Kausalität (Wirkung) angenommen werden kann [46]. Sie betreffen die Frage, (i) wie stark ein empirischer Zusammenhang ist, (ii) ob ein Zusammenhang in verschiedenen Studien repliziert wurde, (iii) ob eine spezifische Exposition ein spezifisches Outcome verursacht, (iv) ob die Exposition dem Outcome zeitlich vorausgeht, (v) ob der Zusammenhang einer Dosis-Wirkungs-Beziehung unterliegt, (vi) ob der Zusammenhang plausibel ist, (vii) ob der Zusammenhang mit dem Stand der Wissenschaft übereinstimmt, (viii) ob der Zusammenhang in Experimenten bestätigt wird und (ix) ob es ähnliche Zusammenhänge gibt, für die eine Kausalität bekannt ist. Demnach ist der Nachweis von Wirkung sehr voraussetzungsreich. In unserer Studie treffen einige Kriterien zu. Zum Beispiel entspricht der von uns gefundene geringe Zusammenhang zwischen der Angebotsnutzung und der kindlichen Entwicklung der Evidenz in anderen Studien (i und ii). Andere Kriterien wiederum treffen nicht oder nur begrenzt zu. Zum Beispiel konnten wir die zeitliche Abfolge von Angebotsnutzung und Entwicklung nicht anhand von echten Längsschnittdaten untersuchen, sondern nur retrospektiv annähern (iv). Eine Dosis-Wirkungs-Beziehung, bei der z. B. die Anzahl der Angebotsbesuche berücksichtigt wird, konnten wir nicht untersuchen (v). Nur wenige Arbeiten haben die Evidenz von Public-Health-Programmen systematisch anhand der Kausalitätskriterien bewertet, z. B. im Hinblick auf Alkoholpreisregulation [47], Notfallhilfe für Opioidkonsumierende [48] oder häusliche Pflege [49]. Ein sinnvoller nächster Schritt in der Bewertung der Frühen Hilfen könnte sein, die Evidenz zu spezifischen Angeboten systematisch zusammenzutragen, in der Gesamtschau zu bewerten, Leerstellen zu identifizieren und eine Forschungsagenda zu entwickeln.

Angesichts der gesellschaftlichen Kosten von langfristig verfestigten Problemen bei sozioökonomisch belasteten Kindern und Familien sollte u. a. durch Nutzung der SEU eine intensivere und differenziertere Prozess- *und* Wirkungsevaluation von Angeboten der Frühen Hilfen vorangetrieben werden [14].

## Fazit

Die Teilnahme an Elternbildung als universellem Angebot der Frühen Hilfen ist mit einer leicht erhöhten Wahrscheinlichkeit eines vollständigen Impfschutzes und einer altersgemäßen Entwicklung verbunden. Die Teilnahme an selektiven Angeboten ist nur mit einem vollständigen Impfschutz verbunden. Allerdings ist die Bewertung selektiver Angebote hinsichtlich der passenden Vergleichsgruppe eine methodische Herausforderung. Es wird mehr Evidenz zu den Wirkungen von Angeboten der Frühen Hilfen benötigt. Die SEU kann unter spezifischen Bedingungen Hinweise zur Wirksamkeit geben. Der Umgang mit Confounding sollte hier besonders berücksichtigt werden.
